# Off–label long acting injectable antipsychotics in real–world clinical practice: a cross-sectional analysis of prescriptive patterns from the STAR Network DEPOT study

**DOI:** 10.1186/s12888-022-04071-2

**Published:** 2022-06-30

**Authors:** Armando D’Agostino, Andrea Aguglia, Corrado Barbui, Francesco Bartoli, Giuseppe Carrà, Simone Cavallotti, Margherita Chirico, Edoardo G. Ostinelli, Caroline Zangani, Giovanni Martinotti, Giovanni Ostuzzi, STAR Network Depot Investigators, Corrado Barbui, Michela Nosè, Marianna Purgato, Giulia Turrini, Giovanni Ostuzzi, Maria Angela Mazzi, Davide Papola, Chiara Gastaldon, Samira Terlizzi, Federico Bertolini, Alberto Piccoli, Mirella Ruggeri, Pasquale De Fazio, Fabio Magliocco, Mariarita Caroleo, Gaetano Raffaele, Armando D’Agostino, Edoardo Giuseppe Ostinelli, Margherita Chirico, Simone Cavallotti, Emilio Bergamelli, Caroline Zangani, Claudio Lucii, Simone Bolognesi, Sara Debolini, Elisa Pierantozzi, Francesco Fargnoli, Maria Del Zanna, Alessandra Giannini, Livia Luccarelli, Alberto De Capua, Pasqua Maria Annese, Massimiliano Cerretini, Fiorella Tozzi, Nadia Magnani, Giuseppe Cardamone, Francesco Bardicchia, Edvige Facchi, Federica Soscia, Spyridon Zotos, Bruno Biancosino, Filippo Zonta, Francesco Pompei, Camilla Callegari, Daniele Zizolfi, Nicola Poloni, Marta Ielmini, Ivano Caselli, Edoardo Giana, Aldo Buzzi, Marcello Diurni, Anna Milano, Emanuele Sani, Roberta Calzolari, Paola Bortolaso, Marco Piccinelli, Sara Cazzamalli, Gabrio Alberini, Silvia Piantanida, Chiara Costantini, Chiara Paronelli, Angela Di Caro, Valentina Moretti, Mauro Gozzi, Chiara D’Ippolito, Silva Veronica Barbanti, Papalini Alessandro, Mariangela Corbo, Giovanni Martinotti, Ornella Campese, Federica Fiori, Marco Lorusso, Lucia Di Capro, Daniela Viceconte, Valerio Mancini, Francesco Suraniti, Maria Salvina Signorelli, Eugenio Rossi, Pasqualino Lupoli, Marco Menchetti, Laura Terzi, Marianna Boso, Paolo Risaro, Giuseppe De Paoli, Cristina Catania, Ilaria Tarricone, Valentina Caretto, Viviana Storbini, Roberta Emiliani, Beatrice Balzarro, Giuseppe Carrà, Francesco Bartoli, Tommaso Tabacchi, Roberto Nava, Adele Bono, Milena Provenzi, Giulia Brambilla, Flora Aspesi, Giulia Trotta, Martina Tremolada, Gloria Castagna, Mattia Bava, Enrica Verrengia, Sara Lucchi, Maria Ginevra Oriani, Michela Barchiesi, Monica Pacetti, Andrea Aguglia, Andrea Amerio, Mario Amore, Gianluca Serafini, Laura Rosa Magni, Giuseppe Rossi, Rossella Beneduce, Giovanni Battista Tura, Laura Laffranchini, Daniele Mastromo, Farida Ferrato, Francesco Restaino, Emiliano Monzani, Matteo Porcellana, Ivan Limosani, Lucio Ghio, Maurizio Ferro, Vincenzo Fricchione Parise, Giovanni Balletta, Lelio Addeo, Elisa De Vivo, Rossella Di Benedetto, Federica Pinna, Bernardo Carpiniello, Mariangela Spano, Marzio Giacomin, Damiano Pecile, Chiara Mattei, Elisabetta Pascolo Fabrici, Sofia Panarello, Giulia Peresson, Claudio Vitucci, Tommaso Bonavigo, Monica Pacetti, Giovanni Perini, Filippo Boschello, Stefania Strizzolo, Francesco Gardellin, Massimo di Giannantonio, Daniele Moretti, Carlo Fizzotti, Edoardo Cossetta, Luana Di Gregorio, Francesca Sozzi, Giancarlo Boncompagni, Daniele La Barbera, Giuseppe Colli, Sabrina Laurenzi, Carmela Calandra, Maria Luca

**Affiliations:** 1 Department of Health Sciences, Università degli Studi di Milano, Milan, Italy; 2 Department of Mental Health, San Paolo Hospital, ASST Santi Paolo e Carlo, Milan, Italy; 3 Department of Neuroscience, Rehabilitation, Ophthalmology, Genetics, Maternal and Child Health (DINOGMI), Section of Psychiatry, University of Genoa, Genoa, Italy; 4 IRCCS Ospedale Policlinico San Martino, Genoa, Italy; 5 WHO Collaborating Centre for Research and Training in Mental Health and Service Evaluation, Department of Neuroscience, Biomedicine and Movement Sciences, Section of Psychiatry, University of Verona, Verona, Italy; 6 Department of Medicine and Surgery, University of Milano Bicocca, Monza, Italy; 7 Department of Psychiatry, University of Oxford, Oxford, UK; 8 Oxford Precision Psychiatry Lab, NIHR Oxford Health Biomedical Research Centre, Oxford, UK; 9 Oxford Health NHS Foundation Trust, Warneford Hospital, Oxford, UK; 10 Department of Neuroscience, Imaging and Clinical Sciences, University “G. d’Annunzio”, Chieti, Italy

**Keywords:** Long-acting injectable antipsychotics, Schizophrenia, Bipolar disorder, Personality disorder, Off-label

## Abstract

**Introduction:**

Information on the off–label use of Long–Acting Injectable (LAI) antipsychotics in the real world is lacking. In this study, we aimed to identify the sociodemographic and clinical features of patients treated with on– vs off–label LAIs and predictors of off–label First– or Second–Generation Antipsychotic (FGA vs. SGA) LAI choice in everyday clinical practice.

**Method:**

In a naturalistic national cohort of 449 patients who initiated LAI treatment in the STAR Network Depot Study, two groups were identified based on off– or on–label prescriptions. A multivariate logistic regression analysis was used to test several clinically relevant variables and identify those associated with the choice of FGA vs SGA prescription in the off–label group.

**Results:**

SGA LAIs were more commonly prescribed in everyday practice, without significant differences in their on– and off–label use. Approximately 1 in 4 patients received an off–label prescription. In the off–label group, the most frequent diagnoses were bipolar disorder (67.5%) or any personality disorder (23.7%). FGA vs SGA LAI choice was significantly associated with BPRS thought disorder (OR = 1.22, CI95% 1.04 to 1.43, *p* = 0.015) and hostility/suspiciousness (OR = 0.83, CI95% 0.71 to 0.97, *p* = 0.017) dimensions. The likelihood of receiving an SGA LAI grew steadily with the increase of the BPRS thought disturbance score. Conversely, a preference towards prescribing an FGA was observed with higher scores at the BPRS hostility/suspiciousness subscale.

**Conclusion:**

Our study is the first to identify predictors of FGA vs SGA choice in patients treated with off–label LAI antipsychotics. Demographic characteristics, i.e. age, sex, and substance/alcohol use co–morbidities did not appear to influence the choice towards FGAs or SGAs. Despite a lack of evidence, clinicians tend to favour FGA over SGA LAIs in bipolar or personality disorder patients with relevant hostility. Further research is needed to evaluate treatment adherence and clinical effectiveness of these prescriptive patterns.

## Introduction

Antipsychotic drugs are classically distinguished in First–Generation (FGA, also called “typical” or “conventional”) and Second–Generation Antipsychotics (SGA, also called “atypical”) based on their mechanism of action and side effects profile [1]. All available formulations are licensed internationally for use in patients diagnosed with Schizophrenia (SCZ), but some regulatory agencies have extended their use to other conditions. Off-label prescription of antipsychotics is very common in clinical practice [2–6], having been estimated to occur in at least one every five patients for oral SGAs [7]. However, reliable data on off-label use of other formulations in clinical practice are lacking. Long–Acting Injectable (LAI) or “depot” formulations have been employed for decades in the treatment of patients with low adherence to oral antipsychotics [7, 8]. International guidelines have recently begun to support their use in first–episode psychosis [6], perhaps leading to an increase in prescription that is likely to also expand their off–label use.

Beyond their established role in the treatment of SCZ, risperidone and aripiprazole LAI are considered a safe and effective alternative to oral medications in the management of Bipolar Disorder (BD) [9]. These two SGA LAIs have been approved by the US Food and Drug Administration (FDA) for maintenance in BD, whereas none have been approved by the European Medical Agency (EMA) for treatment beyond SCZ. Coherently, the Italian Medicines Agency (AIFA) licensed SGA LAI antipsychotics only for SCZ, although three FGA LAIs have long–standing extensions to patients with schizoaffective disorder (haloperidol) and manic states (zuclopenthixol and fluphenazine).

Two recent surveys explored attitudes of Italian psychiatrists towards off–label prescription of both oral and LAI SGAs [10, 11]. The main motivation for prescribing off–label SGAs was the presence of published evidence in the literature (51.5%), followed by a patient’s lack of response to previous on–label treatment (37.1%). In descending order, aripiprazole, olanzapine, risperidone, and paliperidone LAIs were all considered appropriate for the long-term maintenance treatment of BD [10]. Off–label SGA LAI prescription has also been proposed for the treatment of Borderline Personality Disorder (BPD), although no antipsychotic has been approved internationally for this condition and clinicians appear to consider their use inappropriate for these patients [11]. Despite this information, very little is known on the factors that lead clinicians to choose a LAI when commencing treatment in everyday clinical practice. Given the relative poverty of available evidence on LAI use beyond SCZ, and the safety concerns related to off–label antipsychotics, real–world data on prescriptive patterns seem necessary. In particular, the characterization of patients who receive FGA or SGA LAIs off–label may contribute to interpret current practice and to shape future recommendations.

In a large, observational cohort, we have previously shown that patients who receive a novel prescription of any SGA LAI are likely to be younger, occupied, have a diagnosis of either SCZ or BD and have more affective symptoms than those who receive an FGA LAI [12]. Given the relatively sparse knowledge on the everyday off–label use of LAI formulations, we designed an exploratory study in the same cohort with the following objectives:

1. To identify sociodemographic and clinical features of patients treated with on– or off–label LAI prescriptions.
2. To identify predictors of off–label FGA or SGA LAI choice in everyday clinical practice.

## Materials and method

### Study design

This STAR Network Depot study was designed according to the STROBE (STrengthening the Reporting of OBservational studies in Epidemiology) Statement as a multicentre, longitudinal observational study that has been described in detail elsewhere [12]. The protocol was approved by the local Ethics Committees of all participating centres and is publicly available at the Open Science Framework (OSF) online repository (https://osf.io/wt8kx/). All patients who initiated any LAI treatment over a 12–month time span within a consortium comprising 35 territory and university mental health departments in Italy (STAR Network – Servizi Territoriali Associati per la Ricerca), were consecutively screened for inclusion. After screening, each participant was followed up after 6 and 12 months. Inclusion criteria were: (1) participants over 18 years of age, (2) signed informed consent for voluntary participation, and (3) a new prescription of an LAI antipsychotic. Patients who had previously been administered LAIs were only included if the previous one had been suspended for at least 3 months. The recruitment period lasted from December 2015 to May 2017.

For the specific aims of this study, only cross-sectional baseline data were retrieved for all recruited patients. Table 1 shows the diagnostic prescriptive labelling of all LAI antipsychotics retrieved, according to which we compared sociodemographic and clinical variables of two experimental samples (on-label vs off-label). In an exploratory subgroup analysis, we then identified sociodemographic and clinical predictors of FGA vs SGA choice in those who received an off-label prescription.

**Table 1:** List of compounds used as LAI treatment and their on– / off–label indications based on diagnoses in the study cohort

Pharmaceutical Compound	Dosage	Schizophrenia	Bipolar Disorder	Major depression	Obsessive compulsive Disorder	Personality Disorder	Neurodevelopmental Disorder	Neurocognitive Disorder	Other
**Haloperidol Decanoate**^**a**^ **(FGA)**	12,5–300 mg/monthly	**ON LABEL**	**OFF LABEL**	**OFF LABEL**	**OFF LABEL**	**OFF LABEL**	**OFF LABEL**	**OFF LABEL**	**OFF LABEL**
**Zuclopenthixol Decanoate**^**b**^ **(FGA)**	100–600 mg/biweekly	**ON LABEL**	**ON LABEL**	**OFF LABEL**	**OFF LABEL**	**OFF LABEL**	**OFF LABEL**	**OFF LABEL**	**OFF LABEL**
**Fluphenazine Decanoate**^**c**^ **(FGA)**	12,5–100 mg/biweekly	**ON LABEL**	**ON LABEL**	**OFF LABEL**	**OFF LABEL**	**OFF LABEL**	**OFF LABEL**	**OFF LABEL**	**OFF LABEL**
**Risperidone LAI**^**d**^ **(SGA)**	25–50 mg/biweekly	**ON LABEL**	**OFF LABEL**	**OFF LABEL**	**OFF LABEL**	**OFF LABEL**	**OFF LABEL**	**OFF LABEL**	**OFF LABEL**
**Paliperidone Palmitate**^**d**^ **(SGA)**	25–150 mg/monthly	**ON LABEL**	**OFF LABEL**	**OFF LABEL**	**OFF LABEL**	**OFF LABEL**	**OFF LABEL**	**OFF LABEL**	**OFF LABEL**
**Olanzapine Pamoate**^**d**^ **(SGA)**	300–405 mg/monthly	**ON LABEL**	**OFF LABEL**	**OFF LABEL**	**OFF LABEL**	**OFF LABEL**	**OFF LABEL**	**OFF LABEL**	**OFF LABEL**
**Aripiprazole LAI**^**d**^ **(SGA)**	400 mg/monthly	**ON LABEL**	**OFF LABEL**	**OFF LABEL**	**OFF LABEL**	**OFF LABEL**	**OFF LABEL**	**OFF LABEL**	**OFF LABEL**

^a^Haloperidol Decanoate is licensed for the maintenance treatment of Schizophrenia and Schizoaffective Disorder

^b^Zuclopenthixol Decanoate is licensed for acute and chronic Schizophrenia and other dissociative syndromes characterized by symptoms such as hallucination, agitation, psychomotor excitement, hostility, aggressiveness and affective disturbances. Manic phase of manic–depressive psychosis

^c^Fluphenazine decanoate is licensed for the treatment of Schizophrenia and manic syndromes

^d^All SGA LAIs are licensed for the maintenance treatment of adults diagnosed with Schizophrenia

All licensing information refers to the Italian Medicines Agency (Agenzia Italiana del Farmaco, AIFA), *FGA* First–Generation Antipsychotic, *SGA* Second–Generation Antipsychotic

### Clinical assessment

The following sociodemographic and clinical variables were collected upon inclusion: age, sex, education, year of first contact with any psychiatric service, diagnosis, alcohol and other psychoactive substance use, and number and characteristics of hospitalizations in the previous 6 months. At baseline, all patients were assessed with the Italian version of the Brief Psychiatry Rating Scale (BPRS) [13, 14]. A score range from 31 to 40 indicates mild symptoms, from 41 to 52 moderate symptoms, and above 52 severe symptoms [15]. Besides the total score, the following five symptom dimensions were assessed: anxious/depressive symptoms, thought disorder, withdrawal/retardation, hostility/suspiciousness, and activation [16]. The first three dimensions are composed of 4 items each, with each dimension score calculated out of 28; the last two are composed of 3 items each, with each dimension score calculated out of 21.

Additionally, Kemp’s 7-point was employed as a measure of treatment acceptance [17]. Patients were also asked to complete the validated Italian version of the Drug Attitude Inventory 10-items (DAI-10) [18].

### Study population

We previously reported a total of 451 patients (M–F 60.8–39.2%; mean age = 41.6 ± 12.9 years) recruited [12, 19]. In this sample, 251 (55.7%) were diagnosed with SCZ, whereas 81 with BD (18.0%), 74 with schizoaffective disorder (16.4%), 27 with any personality disorder (6.0%) and the rest with a minority of other conditions (3.9%). Given the pragmatic nature of the study, no structured interview was used to confirm diagnoses, which were formulated by participating recruiters based on DSM–5 criteria [20]. For the aims of this study, two groups (on–label vs off–label) were identified by dividing the cohort as shown in Table 2. The World Health Organization broadly defines “off-label” as the use of any medication for an unapproved indication, age group, dosage, duration, or route of administration. Here, off–label use was only considered for unapproved indication at the time of initial prescription. All LAI prescriptions for patients diagnosed within the DSM–5 “Schizophrenia spectrum and other psychotic disorders” clustering [20] were considered on–label. Therefore, patients diagnosed with schizoaffective disorder were considered on–label for any LAI prescription although only haloperidol decanoate is specifically licensed for this condition in Italy.

**Table 2:** Characteristics of patients (*n* = 449) treated with a Long Acting Injection (LAI) drug, divided in on–label and off–label prescription

	ON LABEL	OFF LABEL	SIG
**Diagnosis**			***p*** **= 0.000**
**SCZ (*n*** **= 331)**	331 (100%)	0	
*Schizophrenia*	*251* (100%)	*0*	
*Schizoaffective disorder*	*74* (100%)	*0*	
*Organic Psychosis*	*4* (100%)	*0*	
*Substance-related psychosis*	*2* (100%)	*0*	
**NO-SCZ (*n*** **= 118)**	4 (3.4%)	114 (96.6%)	
*Bipolar Disorder*	*4 (4.9%)*	*77 (95.1%)*	
*OCD*	*0*	*4* (100%)	
*Personality disorder*	*0*	*27* (100%)	
*Neurodevelopment disorder*	*0*	*4* (100%)	
*Neurocognitive disorder*	*0*	*2* (100%)	
Age*	40.97 ± 12.65	44.11 ± 13.18	***p*** **= 0.0334**
**Previous LAI therapy**			*p* = 0.14
Yes (*n* = 135)	107 (79.3%)	28 (20.7%)	
No (*n* = 314)	228 (72.6%)	86 (27.4%)	
**Drug category**			*p* = 0.52
FGA (*n* = 135)	98 (72.6%)	37 (27.4%)	
SGA (*n* = 314)	237 (75.5%)	77 (24.5%)	
**Gender**			***p*** **= 0.044**
Male (*n* = 272)	212 (77.9%)	60 (22.1%)	
Female (*n* = 177)	123 (69.5%)	54 (30.5%)	
**Alcohol**			***p*** **= 0.021**
Yes (*n* = 65)	41 (63.1%)	24 (36.9%)	
No (*n* = 384)	294 (76.6%)	90 (23.4%)	
**Substance Misuse**			*p* = 0.26
Yes (*n* = 90)	63 (70%)	27 (30%)	
No (*n* = 359)	272 (75.8%)	87 (24.2%)	
BPRS total*	50.45 ± 14.66	45.12 ± 14.09	***p*** **= 0.0013**
BPRS anxiety depression*	10.44 ± 4.36	10.88 ± 4.25	*p* = 0.33
BPRS anergy*	9.83 ± 4.27	8.01 ± 3.56	***p*** **= 0.0001**
BPRS thought disturbances*	12.88 ± 5.37	9.91 ± 4.89	***p*** **= 0.0000**
BPRS activation*	7.64 ± 3.35	7.63 ± 3.35	*p* = 0.91
BPRS hostility*	9.68 ± 4.46	8.69 ± 4.40	***p*** **= 0.0474**
Kemp’s 7 total*	4.81 ± 1.44	4.74 ± 1.43	*p* = 0.64
DAI-10 total*	1.78 ± 5.39	2.57 ± 5.24	*p* = 0.15

*SCZ* Schizophrenia Spectrum patients, *NO-SCZ* patients with a diagnosis not included in the schizophrenia spectrum, *OCD* obsessive compulsive disorder, *FGA* First Generation Antipsychotic, *SGA* Second Generation Antipsychotic, *SIG* significance

All reported values are frequencies, except for *(mean ± standard deviation). In bold *p*-values below 0.05

Two patients were excluded from the analysis because the study group could not be assigned due to the missing diagnosis variable. Therefore, the analysed sample includes 449 patients.

### Statistical analysis

We summarised the baseline variables for the recruited sample, as well as for the on–label and off–label groups. To compare the on–label and off–label groups and highlight their differences, we analysed continuous variables with a Mann-Whitney U test, and categorial variables with a Chi^2^ test.

Finally, we ran a multivariate logistic regression analysis to test a number of clinically relevant variables and identify those associated with a different prescription (FGA vs SGA, dependent variable) in the off–label sub-group. The list of the investigated independent variables is sex (female, male), age (continuous), recent use of psychoactive substances (yes, no), recent alcohol use (yes, no), the first treatment with a LAI antipsychotic (yes, no), BPRS subscales (continuous), DAI-10 scale (continuous), and the Kemp’s 7-point scale (continuous). The goodness-of-fit of the resulting model was analysed with a Hosmer-Lemeshow test, while the model was interpreted with the McKelvey-Zavoina pseudo-R^2^. We performed the statistical analyses using Stata 14 [21].

## Results

### On–label vs off–label prescription

Comparative data between the two samples of patients treated with on– and off–label LAIs can be viewed in Table 2. Of 449 patients, 335 (74.6%) belonged to the on–label group and 114 (25.4%) to the off–label one. Almost all the patients in the on–label group (98.8%) presented a SCZ-spectrum diagnosis, whereas most patients in the off–label group had a BD diagnosis (67.5%). The off–label group did not include any patient with a SCZ-spectrum diagnosis. The two groups differed significantly in terms of age and sex, as patients in the on–label group were relatively younger (40.97 ± 12.65 vs 44.11 ± 13.18, *p* < 0.05) and more frequently male (63.3% vs 52.6%, *p* < 0.05). The use of alcohol, but not illicit substances, was found to be more frequent in patients with off–label prescriptions (23% vs 12.2% respectively, *p* < 0.05).

No statistically significant difference was observed between the two groups in terms of FGA vs SGA use. One hundred and seven patients (31.9%) in the on–label group had a previous LAI therapy, compared to 28 (24.6%) in the off–label group.

When BPRS scores were compared between the two groups, total score and anergy, thought disturbances, and hostility dimensions scores were found to significantly differ, being higher in the on–label group. As shown in Table 2, neither DAI-10 scores nor Kemp’s 7-point scales revealed any significant differences between the two groups.

### Off–label FGA vs SGA prescription

**Fig. 1:**
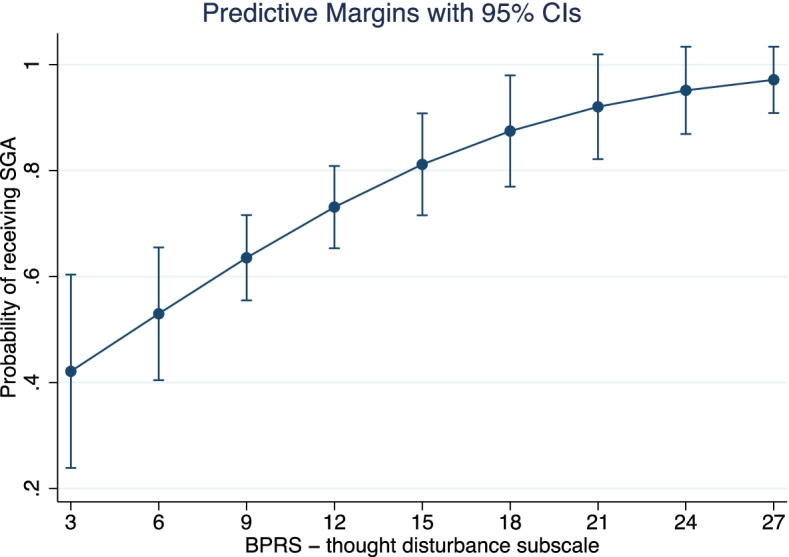
Likelihood of receiving an SGA based on BPRS thought disturbances score. The larger confidence interval suggests greater uncertainty for lower scores

**Fig. 2:**
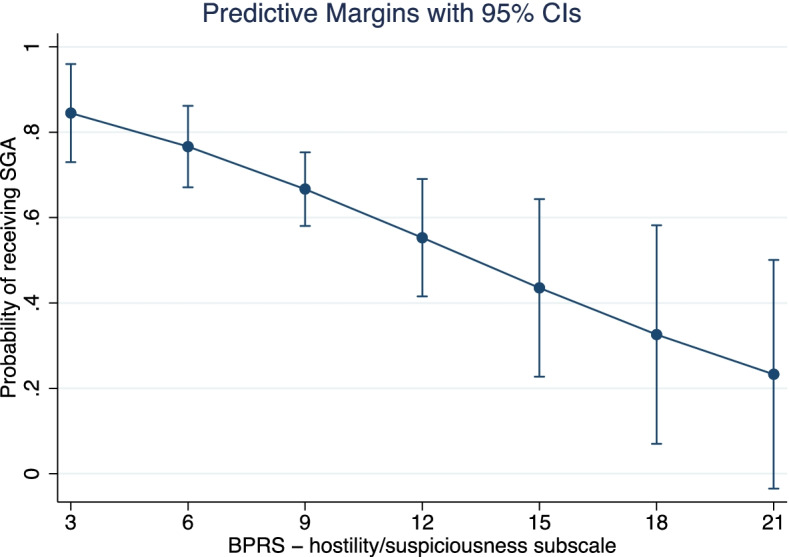
Likelihood of receiving an SGA based on BPRS hostility score. The larger confidence interval suggests greater uncertainty for higher scores

The analysis on the off–label subgroup showed that the preference in choosing between FGA vs SGA LAIs was significantly associated with the BPRS thought disorder and BPRS hostility/suspiciousness dimensions (Table 3), with ORs of 1.22 (CI95% 1.04 to 1.43, *p* = 0.015) and 0.83 (CI95% 0.71 to 0.97, *p* = 0.017), respectively. In this population, the likelihood of receiving an SGA LAI grew steadily with the increase of the BPRS thought disturbance score. Figure 1 shows the predictive margins of SGA prescription for each thought disturbance score, calculated for each observation in the data and then averaged. Conversely, a preference towards prescribing an FGA was observed with higher scores at the BPRS hostility/suspiciousness subscale. Figure 2 shows the predictive margins of FGA prescription for each hostility/suspiciousness score, calculated for each observation in the data and then averaged. The model showed acceptable measures in terms of goodness-of-fit (H-L Chi^2^ *p* = 0.7185) with an MZ pseudo-R^2^ of 31.5%, BIC = 182.214.

**Table 3:** Multivariate analysis comparing the preference in choosing FGA vs SGA drugs in the off-label subgroup

	OR [CI95%]	SIG
Age	0.979 [0.942–1.016]	0.270
Sex		0.356
Male	(ref)	
Female	0.630 [0.236–1.681]	
Substance Misuse		0.271
No	(ref)	
Yes	0.480 [0.130–1.774]	
Alcohol		0.521
No	(ref)	
Yes	1.464 [0.458–4.678]	
First administration		0.359
No	(ref)	
Yes	1.491 [0.502–4.430]	
BPRS anxiety depression	1.102 [0.958–1.267]	0.173
BPRS anergy	0.920 [0.791–1.070]	0.279
BPRS thought disturbances	1.217 [1.039–1.426]	0.015*
BPRS activation	0.913 [0.731–1.140]	0.423
BPRS hostility	0.830 [0.711–0.967]	0.017*
DAI-10 total	1.001 [0.905–1.106]	0.987
Kemp’s 7 total	1.311 [0.896–1.919]	0.896

*P* value of the model = 0.0358, *OR* Odds Ratio, *IC* Confidence Interval, *SIG* significance

## Discussion

The reported study revealed several differences between patients who received on–label and off–label prescriptions of a new LAI antipsychotic. In the off–label group, BD was the most frequent diagnosis, followed by personality disorders. Since all the included LAIs are licenced for SCZ, all patients with a SCZ–spectrum diagnosis were included in the on–label group. This may have contributed to the observed differences between the two samples. For example, the difference observed in terms of alcohol use between the on– and off–label groups could be ascribed to relatively lower alcohol consumption in SCZ patients compared to those with mood and personality disorders [21]. However, alcohol use is strongly associated with impulsivity and behavioural abnormalities [22] and might encourage the choice of a LAI treatment, albeit off–label. Likewise, the greater intensity of symptoms observed in the on–label group might reflect a relatively worse psychopathology in SCZ patients compared to those with BD and personality disorders when a new LAI is prescribed. Several sub-items – anergy, hostility, and thought disturbances – were higher in the on–label group compared to the off–label one. Indeed, compared to those with other diagnoses, SCZ patients typically present more negative symptoms, thought disturbances and hostility driven by a substantial lack of insight.

Although the BPRS may not fully capture the extent of clinical symptoms presented by patients with mood and personality disorders, our findings suggest off–label LAIs are prescribed in these patients despite a relatively lower intensity of psychopathology compared to SCZ patients who begin a new on–label LAI. Previous studies have shown that LAI treatment in BD is a viable option for patients with low treatment adherence or an unstable illness with predominant manic recurrences [23]. A panel of experts recently suggested that BD patients with co–morbid substance use disorder, family history of bipolar illness, and use of multiple medications may be particularly good candidates for LAI antipsychotic treatment [24].

Of note, females were relatively more present in the off–label group, perhaps reflecting the epidemiology of clinical diagnoses in this sample. Indeed, female sex is relatively more represented in cohorts of patients with mood and personality disorders compared to SCZ [25, 26]. The finding of a slightly older mean age in the off–label group could suggest that off–label LAI prescription is delayed in these patients’ clinical course, perhaps due to uncertainty on safety and effectiveness.

Our study is also the first to identify sociodemographic, clinical features and predictors of FGA vs SGA choice in patients treated with off–label LAI antipsychotics. In general, we found that SGA LAIs were more commonly prescribed in everyday practice, without significant differences in their on– and off–label use. In the off–label group, we identified predictors of FGA or SGA LAI choice in everyday clinical practice. Among several tested variables, demographic characteristics (i.e., age, sex) and comorbidities, such as alcohol and substance use, seem not to influence the choice towards FGAs or SGAs. A higher mean score in the hostility/suspiciousness symptom dimension was associated with an increased likelihood of receiving an FGA compared to an SGA LAI prescription. Hostility implies a tendency to feel anger towards people, which has been associated with aggressiveness in a variety of mental disorders [27]. Hence, this association might underline the clinical tendency to judge FGAs more efficient than SGAs for the treatment of aggressiveness, with the latter preferred to address mood and positive symptoms. Indeed, LAIs are known to significantly reduce the hostility, aggressiveness, and frequency of violent episodes in SCZ spectrum diagnoses [28]. However, the literature on this topic is poor and recent studies failed to show significant differences between FGAs and SGAs [29, 30]. If the choice of an FGA is supported by well-established practical experience and economic consideration, the tolerability profile might favour SGAs. Indeed, a recent retrospective chart review study of 157 cases with SCZ spectrum diagnoses suggested prescriber choice should be guided by factors such as side–effect profile, patient acceptability and price [31]. Nonetheless, a class profile of tolerability could be misleading because specific compounds have been associated with very different side effect profiles [32]. In the same cohort, we have previously shown that clinicians are more inclined to prescribe paliperidone palmitate than aripiprazole monohydrate to subjects with higher symptom severity [31], although the latter might be superior in terms of tolerability and healthcare costs [33, 34].

Notably, neither the clinician–rated adherence to treatment nor the patient–rated attitude towards medication differentiated on– and off–label prescription groups. Likewise, neither appeared to predict the choice of administering FGA vs SGA in the off–label group. Taken together, these findings suggest that low adherence and negative attitude towards medication are defining aspects of patients who receive a new LAI prescription, independent of its licensing or generation.

### Study limitations

This study has several limitations. First, we only examined cross-sectional data at the time of a novel LAI prescription, so efficacy and tolerability could not be evaluated. Future analyses of longitudinal modifications will be useful to have an insight of the course of the off–label prescriptions in our cohort. Second, clinical diagnoses were not confirmed through a structured interview, so some variability can be expected to have occurred across sites. Moreover, a large group of patients with diagnoses other than SCZ were considered on–label if their diagnosis fell within the DSM-5 “Schizophrenia spectrum and other psychotic disorders” clustering. In particular, 74 patients with schizoaffective disorder were considered on–label although no SGA LAI has specifically been licensed for this diagnosis. Nonetheless, we chose to include patients with this controversial diagnosis in a SCZ grouping, in line with both DSM–5 [20] and ICD–11 [35]. Third, it was not possible to ascertain whether and to what extent legal practices for the off–label prescription of drugs were employed, and if adequate information was provided to the patients. Further studies to assess this issue might be relevant, considering possible risks of the frequent use of off–label medications in vulnerable populations of patients with severe mental disorders. Fourth, no information was available on the mood episode or dominant polarity of BD and schizoaffective disorder patients, which might have influenced the prescriber’s choice of FGA vs SGA LAI. Finally, the characteristics of centres involved in the Italian STAR Network were heterogeneous and different local availability of drug formulations might have affected the choice of treatment.

## Conclusion

Both FGA and SGA LAIs are frequently prescribed off–label in real–world clinical practice, particularly in people diagnosed with BD and personality disorders. Although no previous evidence supports a larger benefit of FGA LAIs when addressing hostility, clinicians tend to privilege them over newer compounds when initiating off–label LAI treatment, whereas SGA LAIs are generally preferred in patients with more severe thought disturbances. Future analyses on the study cohort follow-up data will be fundamental to evaluate treatment adherence and the clinical effectiveness of these prescriptive choices.

## Data Availability

The full dataset is available from the Dryad Digital Repository, doi:10. 5061/dryad.q49p6d8.
